# Endovascular Repair of Ruptured Abdominal Aortic Aneurysms Using the Endurant™ Endograft

**DOI:** 10.3390/jcm13175282

**Published:** 2024-09-06

**Authors:** Petroula Nana, George Volakakis, Konstantinos Spanos, George Kouvelos, Metaxia Bareka, Eleni Arnaoutoglou, Athanasios Giannoukas, Miltiadis Matsagkas

**Affiliations:** 1 Department of Vascular Surgery, Larissa University Hospital, Faculty of Medicine, University of Thessaly, 41110 Larissa, Greece; geo-vol@hotmail.com (G.V.); spanos.kon@gmail.com (K.S.); geokouv@gmail.com (G.K.); agiannoukas@hotmail.com (A.G.); milmats@gmail.com (M.M.); 2 German Aortic Centre, Department of Vascular Medicine, University Heart and Vascular Centre, UKE Hamburg, 20246 Hamburg, Germany; 3 Department of Anaesthesiology, Larissa University Hospital, Faculty of Medicine, University of Thessaly, 41110 Larissa, Greece; barekametaxia@hotmail.com (M.B.); earnaout@gmail.com (E.A.)

**Keywords:** abdominal aortic aneurysm, rupture, endovascular repair, parallel grafts, Endurant

## Abstract

**Background:** Endovascular aortic aneurysm repair (EVAR) represents a valid treatment modality for ruptured abdominal aortic aneurysms (rAAAs). This study aimed to present rAAA outcomes treated by EVAR using the Endurant endograft. **Methods:** A single-center retrospective analysis of consecutive patients treated with standard EVAR (sEVAR) or parallel graft (PG)-EVAR for infra- or juxta/para-renal rAAA using the Endurant endograft (1 January 2008–31 December 2023) was undertaken. The primary outcomes were technical success, mortality, and reintervention. Follow-up outcomes, including survival and freedom from reintervention, were assessed using Kaplan–Meier estimates. **Results:** Eighty-eight patients were included (87.5% sEVAR and 12.5% PG-EVAR). The mean aneurysm diameter was 73.3 ± 19.3 mm (71.4 ± 22.2 mm sEVAR and 81.7 ± 33.0 mm PG-EVAR). Among 77 patients receiving sEVAR, 26 (33.8%) received an aorto-uni-iliac device. All PG-EVAR patients were managed with bifurcated devices, one receiving a single PG, seven double PGS, and three triple PGs. Technical success was 98.8% (100.0% sEVAR and 90.9% PG-EVAR). The 30-day mortality was 47.2% (50.7% sEVAR and 27.3% PG-EVAR), with nine (10.2%) deaths recorded on the table. The mean time of follow-up was 13 ± 9 months. After excluding 30-day deaths, the estimated survival was 75.5% (standard error (SE) 6.9%) at 24 months. The estimated freedom from reintervention was 89.7% (SE 5.7%) at 24 months. Only one endoleak type Ia event was recorded during follow-up. **Conclusions:** Endurant showed high technical success rates and low rates of endoleak type Ia events and reinterventions, despite the emergent setting of repair. rAAA is still a highly fatal condition within 30 days, with an acceptable mid-term survival of 30-day survivors at 75.5%.

## 1. Introduction

A ruptured abdominal aortic aneurysm (rAAA) is a medical emergency, related with >50% mortality, even before a patient’s admission to the hospital [1]. After immediate repair, early mortality rates have been recorded up to 35%, although with decreasing rates during the last decade [2,3,4]. The wide use of endovascular aneurysm repair (EVAR), paired with standardized perioperative care protocols and referral to dedicated aortic centers, seems to improve rAAA repair outcomes [2]. However, complex anatomies prevent the use of standard EVAR, due to restrictions mainly applying to the proximal sealing zone [5]. Off-the-shelf techniques such as surgeon-modified grafts and the parallel graft (PG) technique have been used to address complex rAAAs [6,7]. The use of commercially available endografts and covered stents in the PG technique provide an additional benefit, taking into consideration the less technically demanding procedure compared to endograft modifications [6,7]. 

Regarding the choice of endograft used for emergent EVAR, currently available off-the-shelf devices provide various options, depending on the patient’s anatomy, the surgeon’s preference, and the endografts’ availability [8]. However, it has been shown that rAAA anatomy is more challenging than the one met in elective cases [8]. Previously published experience with the Endurant endograft (Medtronic, Santa Rosa, CA, USA) showed high technical success without type Ia endoleaks at 30 days, low perioperative morbidity, and null mortality in elective patients with challenging proximal anatomy [9,10]. Patients managed with the PG technique using the Endurant device showed also high technical success, acceptable type Ia, and low mortality [11]. However, data reporting on the use of the Endurant exclusively in an emergent setting, either for EVAR or PG-EVAR, are limited [12].

The aim of this study is to present a single tertiary center experience of rAAA treated by EVAR or PG-EVAR using the Endurant endograft.

## 2. Materials and Methods

### 2.1. Study Design 

A single-center retrospective analysis of consecutive patients presenting with infrarenal or juxta- and para-renal rAAA and treated with EVAR or PG-EVAR using exclusively the Endurant endograft, from 1 January 2008 to 31 December 2023, was undertaken. The Strengthening the Reporting of Observational Studies in Epidemiology (STROBE) guidelines were followed [13]. The study complied with the Declaration of Helsinki and was considered exempt from ethical approval, due to its retrospective design and use of anonymized data.

### 2.2. Study Cohort 

Only patients with rAAA managed with the Endurant endograft were considered eligible, regardless of the use of bifurcated or aorto-uni-iliac (AUI) device. The patients were managed immediately upon their arrival at the hospital in case of hemodynamic instability and after no more than an hour in stable patients. In many patients transferred from other regional hospitals, a review of the computed tomography angiography (CTA) was available before patients’ arrival, while in those without CTA, imaging was undertaken immediately upon their arrival. Patients with an infra-renal neck ≥ 10 mm of length and diameter ≤ 32 mm were considered for standard EVAR, while patients with a compromised proximal infra-renal sealing zone (length < 10 mm and diameter > 32 mm) were considered candidates for PG-EVAR [14]. Conical morphology was not a criterion for exclusion for standard EVAR, according to previously published criteria [14].

Sizing and planning were performed by experienced vascular surgeons, based on the preoperative CTA, using a workstation with dedicated software (3Mensio, Medical Imaging B.V., Bilthoven, The Netherlands). All EVAR procedures were performed in an adequately equipped operating room, using a moveable radiolucent surgical table (2006–2019: Maquet, Getinge Group, Rastatt, Germany and 2019–2023: Stille Surgical, Inc., Torshälla, Sweden) and a mobile digital angiographic system (2006–2019: Philips BV Endura, Philips Medical Systems, Release 2.2.3, Amsterdam, The Netherlands and 2019–2023: Ziehm Vision RFD Hybrid Edition, Ziehm Imaging, Nuremberg, Germany). All procedures were performed by experienced vascular surgeons. A dedicated peri-operative protocol was used in all patients with rAAA, interpreting the principles of permissive hypotension, local anesthesia, and use of an aortic occlusion balloon in hemodynamically unstable cases [15]. 

Patients managed with any other device were excluded from this analysis (Supplementary Figure S1). Patients managed for type Ib or IIIa endoleaks only with limb extension, limb relining, or only proximal cuff (7 cases) were also excluded due to the lower complexity of the procedure. Patients that arrived in the operating room but died before complete endograft deployment were omitted (1 case). Symptomatic patients without any imaging sign of aortic rupture were not included. These patients were not managed immediately but within the same hospitalization (24–48 h) if no sign of rupture appeared during hospitalization. 

### 2.3. Device and Technical Details 

The bifurcated Endurant device was preferably used. However, the aorto-uni-iliac (AUI) system was also applied in some cases, depending on distal landing zones, the surgeon’s preference, and device availability [16]. AUI systems were mainly preferred during the department’s early experience. In these cases, an iliac occluder was placed in the contralateral common iliac artery and an 8 mm PTFE graft was used (W. L. Gore & Associates, Flagstaff, AZ, USA) for the femoral–femoral bypass.

Regarding the Endurant characteristics, the stents are M/Z-shaped, made of nitinol, and sewn to a polyester fabric graft, while proximal sealing is secured with active suprarenal fixation, having barbs in the suprarenal stent. According to the current instructions for use, Endurant can be applied in patients with a proximal infrarenal neck length equal to or more than 10 mm and an angle up to 75°. The bifurcated body requires one or two iliac extensions, and the contralateral gate is cannulated in standard fashion. The delivery system is sheath-less, with a profile of up to 20 Fr for the main body [16]. The PG-EVAR technique has been previously described [17]. Balloon-expandable covered stents, including the BeGraft Peripheral, BeGraft Plus (Bentley, Hechingen, Germany), and VBX (W.L. Gore & Associates, Flagstaff, AZ, USA), were used for visceral vessel preservation. Bilateral common femoral artery access was performed using cut-down in all cases. In PG-EVAR patients, axillary or brachial access was used for parallel graft advancement, depending on the number of target vessels. 

### 2.4. Data Collection 

Pre-, intra-, and post-operative information were collected by an experienced team consisting of vascular surgeons and a study nurse, after pseudonymization, in a dedicated database. Regarding follow-up data, the surveillance protocol for rAAA included computed tomography angiography (CTA) before discharge, at 1–3 months, 12 months, and yearly thereafter. However, imaging surveillance intervals and the type of imaging (e.g., duplex ultrasound (DUS)) could be modified, depending on the imaging findings or the patients’ status and renal function. Available data during follow-up were also added into the same database. Patients who did not attend two subsequent scheduled appointments were considered lost to follow-up.

### 2.5. Definitions

Regarding definitions, the reporting standards on EVAR were used for patients managed with standard bifurcated or AUI devices [18]. For patients managed with the PG-EVAR, the technical success was defined by the Society of Vascular Surgery reporting standards for endovascular aortic repair of aneurysms involving the renovisceral aorta [19]. In these cases, technical success was defined as the successful introduction and deployment of the main device at the intended landing zone, with target vessel (TV) preservation, absence of surgical conversion or mortality, and absence of endograft or parallel graft occlusion or stenosis [19]. The presence of type I or III endoleaks at the completion angiography was not considered a technical failure, as many of these endoleaks tend to be self-limiting within 30 days [19]. Similarly, acute kidney injury (AKI) was defined according to the latest reporting standards regardless of the underlying procedure (EVAR or PG-EVAR) [18].

### 2.6. Outcomes

The primary outcomes were technical success, mortality, and reintervention at 30 days. Follow-up outcomes, including the survival, freedom from reintervention, and endoleak type I, were considered secondary. 

### 2.7. Statistical Analysis

Continuous data were reported as the mean ± standard deviation. Categorical data were expressed as absolute numbers and the percentage of prevalence (%) in the study cohort. No comparisons were attempted between groups. Univariate and multivariate regression analyses would be performed only if the number of adverse events exceeded 10 according to the rule of thumb, leading to an analysis only of factors related to 30-day mortality. The study period was divided into early (2008–2015) and late (2016–2023) and the impact of experience on 30-day outcomes was also examined. Only parameters providing a statistical significance through the univariate analysis would be imputed in the multivariate regression analysis. Follow-up parameters were assessed using Kaplan–Meier estimates. The *p* value was considered significant when it was <0.05 (two-tailed analysis). The sample size was allowed to vary based on the analysis and no imputation of missing data in early or in follow-up analyses was performed, while missing baseline categorical and continuous variables were infrequent (<5%). Statistical analysis was performed by SPSS 22.0 for Windows software (IBM Corp, Armonk, NY, USA). 

## 3. Results

### 3.1. Patient Cohort 

In total, 88 patients were managed with the Endurant device. Among them, 77 (87.5%) needed standard EVAR and the remaining 11 (12.5%) were managed with at least one parallel graft to preserve a renal or visceral artery. Females represented 12.5% (11) of the cohort and the mean age was 73.4 ± 5.4 years. The baseline characteristics of the cohort are presented in Table 1. Coronary artery disease was reported in 31 (35.2%) patients; 6 (54.5%) of them were in the PG-EVAR group.

The mean aneurysm diameter was 73.3 ± 19.3 mm for the total cohort; 71.4 ± 22.2 mm in the standard EVAR group; and 81.7 ± 33.0 mm in the PG-EVAR group. Twenty-four (27.3%) patients were presented with a free rupture, with three (27.3%) belonging to the PG-EVAR group. All patients managed with standard EVAR presented with an infrarenal aneurysm; presenting at least 10 mm of appropriate neck length. Regarding the PG group, four (36.4%) patients were managed for a juxtarenal aneurysm and seven (63.6%) for a pararenal aneurysm. Among the total cohort, ten (11.4%) patients had a type Ia endoleak after previous EVAR, while no patient had a history of previous open surgical repair. Table 2 presents the anatomic details of the cohort and the standard EVAR and PG groups. 

Regarding repairs, among these 77 patients managed with standard EVAR, 26 (33.8%) received an AUI device and a femoral–femoral bypass. All PG-EVAR patients were managed with bifurcated devices while all target vessels were bridged using a BeGraft peripheral stent; except four renal arteries and one superior mesenteric artery (SMA), which were bridged with a VBX stent. Seven patients received double parallel grafts for renal artery preservation and one double parallel graft for the preservation of the right renal artery and SMA, as the patient already presented with left renal artery occlusion. Triple parallel grafts were performed in three patients for renal and SMA preservation. Only one patient received a single parallel graft for SMA preservation. In this case, the patient was managed for a 105 mm juxta-renal aneurysm due to endoleak type Ia after previous EVAR. Renal artery catheterization was attempted but failed due to the implication of the struts of the previous endografts just in front of both renal artery ostia. Renal sacrification was decided intra-operatively. 

**Figure 1:**
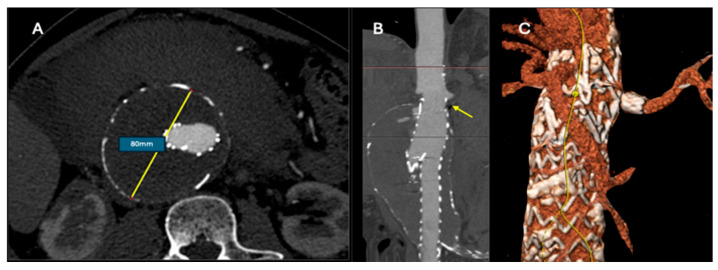
PG-EVAR for ruptured AAA after previous EVAR. Eleven patients were managed with PG-EVAR. Among them, a patient with an 80 mm juxta-renal aneurysm after previous EVAR was treated with a single parallel graft targeting the preservation of the left renal artery (**A**,**B**). The short infrarenal neck did not permit the application of standard EVAR ((**B**); yellow arrow). The 36-month CTA confirmed the favorable technical outcome with no endoleak and a patent target vessel (**C**).

Local anesthesia with monitored anesthesia care (MAC) was initially used in all patients; in 56 (63.6%) patients, it was used until the completion of the procedure. In the remaining 32 patients, a conversion to general anesthesia was decided intra-operatively due to patients’ agitation or hemodynamic collapse. Two patients were managed with the Resuscitative Endovascular Balloon Occlusion of the Aorta (REBOA) technique; both were standard EVAR cases. Nine (10.2%) patients died on table with the completion of the procedure, all managed due to free rupture and none of them receiving a parallel graft (Figure 1). Technical success was achieved in 87 (98.8%) cases, except for the case where renal catheterization failed. Intra-operative information is presented in Table 3.

### 3.2. Early Postoperative Findings 

Post-operatively, 48 (60.8%, 48/79 patients that survived the operation) patients remained under surveillance in the intensive care unit (ICU), 43 (63.2%, 43/68) from the standard EVAR group and 5 (45.5%, 5/11) from the PG group, while the remaining were transferred to the ward under close surveillance. The median duration of ICU stay was 3 days (IQR 1.5–5 days), 3 days (IQR 1.5–11 days) for the standard EVAR group, and 2 days (IQR 1.5–5 days) for the PG group. 

In total, 42 (47.2%) patients died within the initial 30 days; 39 (50.7%) from the standard EVAR group and 3 (27.3%) from the PG group. Three patients presented AKI needing dialysis, one patient suffered a transmural myocardial infarction, and two presented pulmonary insufficiency needing prolonged intubation; one was related to COVID infection. The remaining patients died due to multiorgan failure related to the hemorrhagic shock. The multivariate regression analysis revealed no independently related factor; free rupture presented a tendency (*p* = 0.06) but did not provide significance. 

Reinterventions were performed in three patients: all from the standard EVAR group. Two reinterventions were related to access and one was related to a type Ia endoleak, managed with a successful aortic cuff extension. The thirty-day outcomes are presented in Table 4. 

### 3.3. Follow-Up Outcomes 

**Figure 2:**
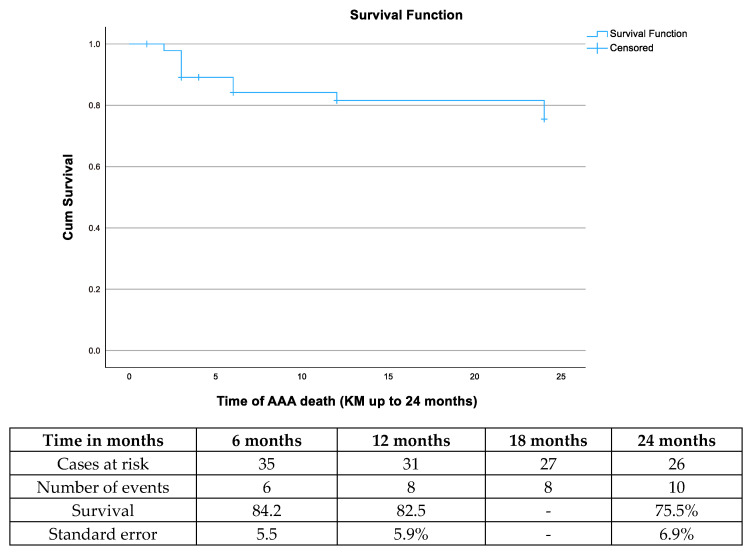
The estimated survival after endovascular aortic aneurysm repair for a ruptured abdominal aortic aneurysm among survivors after 30 days.

The mean time of follow-up was 13 ± 9 months, 14 ± 9 months in the standard EVAR group, and 17 ± 15 months in the PG group. After excluding the patients that died within the initial 30 days, the estimated survival was 81.5% (standard error (SE): 5.9%) at 12 months and 75.5% (SE: 6.9%) at 24 months with nine additional deaths in the standard EVAR group and one in the PG group (Figure 2). Among them, two events were aneurysm-related (4.3%, 2/46; both in the standard EVAR group); one occurred 50 days after the procedure in a patient with extended hospitalization and in-hospital infection and a second one due to aneurysm rupture related to a type Ia endoleak. For the remaining deaths, four were related to cancer, two to stroke, and two to cardiac causes (one related to arrythmia and one to myocardial infarction). 

**Figure 3:**
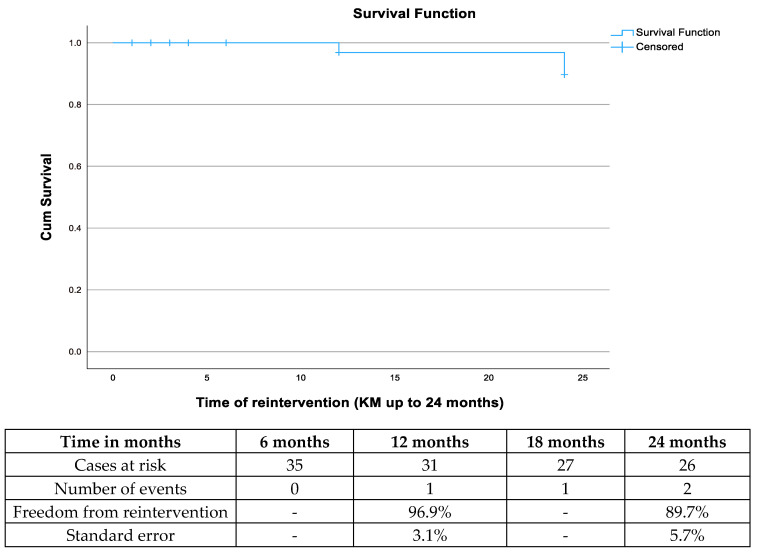
Freedom from reintervention after endovascular aortic aneurysm repair for a ruptured abdominal aortic aneurysm among survivors after 30 days.

Regarding endoleaks, only one event of type Ia endoleak was recorded. No type Ib or III endoleaks were detected during follow-up. The estimated freedom from reintervention was 96.9% (standard error (SE): 3.1 SE%) at 12 months and 89.7% (SE: 5.7%) at 24 months (Figure 3). In total, three reinterventions were reported, all in the standard EVAR group. One coiling related to a type II endoleak and a sac increase of 5 mm within 6 months and one iliac limb occlusion managed with mechanical thrombectomy and relining. The last reintervention was related to pseudoaneurysm formation in a left femoral access site managed with open reconstruction. No parallel graft occlusion or stenosis was detected during follow-up and no reintervention was performed.

## 4. Discussion

Despite the outcomes of randomized trials comparing the use of EVAR vs. open surgical repair (OSR) under elective, urgent, and emergent settings during the previous decade, EVAR has expanded its targeted population and currently represents the main approach for rAAA management, due to the related lower mortality and morbidity during the post-operative period [20,21,22,23,24,25]. Regardless of the evolvement in both the technique and materials, rAAA still remains a highly fatal event, as also reported in the current study, with more than 30% of patients dying within the initial 30 days [26,27]. However, in technical aspects, Endurant performed with high technical success, in both standard and complex EVAR procedures, and a low reintervention rate, even in patients managed with PG-EVAR during the early and mid-term follow-up. Type Ia endoleak was rather a sporadic phenomenon within the cohort. Among various, currently available devices in the market, Endurant has been related to highly encouraging outcomes in elective, urgent, and emergent settings in real-world studies [9,28].

Current recommendations suggest the use of EVAR over OSR, depending on the patient’s anatomy, in cases presenting with rAAA [15]. However, the last decade, complex endovascular procedures have been performed using off-the-shelf, surgeon-modified grafts and the PG technique in urgent settings with very encouraging outcomes, especially for symptomatic non-rupture cases [29,30,31]. Despite the fact that the parallel graft technique has received important criticism in terms of durability and type Ia endoleaks, it represents a valuable off-the-shelf solution in urgent procedures associated with high technical success and acceptable mortality [32,33]. According to the current analysis and despite the small sample size of 11 complex cases, PG-EVAR with the Endurant platform seems to be a reliable approach in urgent settings, with technical success at 90% and high target vessel patency during the available follow-up, even if patients with previous EVAR were included [33]. Despite the fact that current recommendations suggest the use of up to two PGs; aligning with Medtronic’s IFU for Endurant as the main endograft in PG cases, three triple PG cases were included in this analysis, without any impact on technical aspects, including the presence of type Ia endoleaks and TV patency [15].

Technical success was high for the total cohort, over 98%, while only one technical failure was recorded in one patient with previous EVAR presenting significant renal artery stenosis and needing triple PG-EVAR [9,34]. The presence of previous aortic repair, endovascular or open, has been related to higher complexity and lower technical success rates according to the current literature on fenestrated and branched endovascular repair after previous aortic intervention [35,36]. The high technical success seems to sustain through the early and mid-term follow-up with only one case related to type Ia, leading to one aorta-related mortality event during the mid-term follow-up. Pre-operative CTA is a mandatory step to reassure technical outcomes, and all our patients underwent a diagnostic CTA before the procedure, not only to confirm diagnosis but also to permit safe planning of the upcoming procedure after a detailed evaluation of the aortic anatomy. Despite the fact that rAAA cases present far more complex anatomy in terms of neck hostility, conicity, and angulation, Endurant seems to be able to adapt under these extreme anatomic demands, providing low rates of high-flow endoleaks [37]. Another parameter affecting the PG-EVAR technical outcomes with Endurant has to do with the combination of the endograft with appropriate bridging stents. Our experience is mainly represented by the combination of Endurant and BeGraft with favorable outcomes in terms of the absence of gutters and TV patency, even in these emergent circumstances [38,39,40]. However, Endurant’s conformability around the parallel grafts, in addition to an appropriate oversizing at 30%, seems to favor technical outcomes with other combinations of bridging stents, as the Advanta V12 [38].

The mortality was relatively higher than would be anticipated, at 45% during the initial 30 days. Despite the fact that in all cases the procedure was completed, nine patients never left the operating theater due to severe hemodynamic instability, showing that the underlying aortic rupture is the leading factor affecting clinical outcomes, regardless of the technical success of the procedure. None of the on-table deaths were within the PG-EVAR group, a noteworthy fact potentially related to better patient selection and the fact that the most experienced surgeons of the department participated in the management of these cases. However, the findings of the analysis should be evaluated under the presence of a free rupture in almost 27% of patients and the fact that patients from the center’s early experience in EVAR were also included [25,41]. Local ethics may affect decision making, offering treatment in unstable elder patients that would be potentially excluded from operative management under other ethical and social circumstances. In addition, the pre-operative comorbidities’ profile of the patients should be taken into consideration when evaluating the early mortality outcomes, with 30% of them having a known history of coronary artery disease and 25% presenting with chronic obstructive pulmonary disease (COPD). All these factors added to the hemodynamic instability and the acute nature of rAAA may be able to justify the mortality-related findings [42]. Recent data evaluating the mortality after EVAR for rAAA showed a significant decrease in early mortality through a timespan of 15 years; inversely proportional to increasing experience [43].

However, patients that survive this critical early period seem to present acceptable survival rates at least within the mid-term follow-up, with mortality causes being non-related to any aortic pathology [10,43]. The loss to follow-up, though, should be acknowledged, and it affected 30% of the cohort. However, such rates are not uncommon within patients managed endovascularly and may be affected by multiple parameters, including the social and economic status of the patient [44]. Reinterventions and endoleaks were sporadic during follow-up, and they could not be directly related to mortality after 30 days, showing that the Endurant provides the needed stability in time, to prevent fatal aortic adverse events. However, previous studies showed that EVAR for rAAA was related to high reintervention rates during the mid-term follow-up [45]. Longer follow-up and larger cohort studies, especially of urgent and emergent populations, are of major importance to provide robust conclusions on the durability of EVAR in rAAA.

### Limitations

The main limitations of the study are its retrospective nature, small sample size, and limited time of available follow-up. Despite the higher number of patients treated for rAAA, a few of them, managed only with Endurant components, were excluded to increase the homogeneity of the cohort. Patients from the center’s very early experience (first endovascularly managed rAAA was performed in 2011; start year of this study) were included and may have affected our findings. However, patients managed with AUI devices were included, as in our opinion the challenges during the repair were not highly different than these confronted in standard bifurcated EVAR cases. Direct comparisons between the standard EVAR and PG-EVAR groups were not attempted, not only to avoid type I and II statistical errors and extreme outliers in regression analyses, arising from small group comparisons, but also and more importantly as the groups presented an a priori significant difference in terms of anatomy and the complexity of repair. Specific data on the time from admission to the beginning of the procedure were not available and this parameter was not further analyzed but it has been detected in the past as a factor affecting early mortality [46]. Regression analyses were performed only for 30-day mortality and not for technical success and reinterventions at 30 days, due to the limited number of adverse events, which would not permit safe assessment. Despite the fact that a 15-year experience with the Endurant endograft is presented in this study, the mean follow-up was restricted to 12 months—a fact related mainly to two parameters; first, the 45% 30-day mortality rate, and second, the associated loss to follow-up, which was estimated at 30% among patients that survived 30 days after the procedure. The lack of robust long-term data should be taken into consideration during the evaluation of the follow-up findings of the current analysis. However, the standard error in Kaplan–Meier curves was below 10% up to 24 months, providing a rather safe assessment until that time-point. Longer-term data are of major importance and further studies are needed to provide more robust conclusions.

## 5. Conclusions

Endurant showed high technical success and low type Ia endoleak and reintervention rates at 30-day and 24-month follow-ups, despite the emergent setting of repair and the presence of 27% free ruptures. rAAA is still a highly fatal condition within 30 days, while the mid-term survival of 30-day survivors was 75.5%.

## Figures and Tables

**Table 1:** Baseline characteristics. The total cohort’s characteristics as well as the per type of repair baseline characteristics and comorbidities.

Baseline Characteristics	Total Cohort (88 Patients)	Standard EVAR (77 Patients)	Parallel Graft EVAR (11 Patients)
Age (years)	73.4 ± 5.4	74.0 ± 5.9	69.7 ± 8.7
Males	77 (87.5%)	67 (87.0%)	10 (90.9%)
Hypertension	69 (78.4%)	58 (75.3%)	11 (100.0%)
Diabetes mellitus	19 (21.6%)	19 (24.7%)	0 (0.0%)
Dyslipidemia	49 (55.7%)	39 (50.6%)	10 (90.9%)
Tobacco use	47 (53.4%)	42 (54.5%)	5 (45.5%)
Coronary artery disease	31 (35.2%)	25 (32.5%)	6 (54.5%)
Chronic kidney disease	6 (6.8%)	3 (3.9%)	3 (27.3%)
Atrial fibrillation	7 (8.0%)	5 (6.5%)	2 (18.2%)
COPD	22 (25.0%)	19 (24.7%)	3 (27.3%)
Stroke	5 (5.6%)	4 (5.1%)	1 (9.1%)

COPD: chronic obstructive pulmonary disease; EVAR: endovascular abdominal aortic aneurysm repair.

**Table 2:** Anatomy and previous repairs. Anatomic details of the total cohort, as well as per type of repair.

Anatomic Details	Total Cohort (88 Patients)	Standard EVAR (77 Patients)	Parallel Graft EVAR (11 Patients)
Aneurysm diameter (mm)	73.3 ± 19.3	71.4 ± 22.2	81.7 ± 33.0
Free rupture	24 (27.3%)	(27.3%)	3 (27.3%)
Aneurysm extension			
Infrarenal	77 (87.5%)	77 (100.0%)	0 (0.0%)
Juxtarenal	4 (4.5%)	0 (0.0%)	4 (36.4%)
Pararenal	7 (8.0%)	0 (0.0%)	7 (63.6%)
Previous EVAR	10 (11.4%)	5 (6.5%)	5 (45.5%)
Endoleak type Ia	8 (9.1%)	3 (3.9%)	5 (45.5%)
Endoleak type Ib associated to neck dilation/graft migration	2 (2.3%)	2 (2.6%)	0 (0.0%)
Endoleak type II	0 (0.0%)	0 (0.0%)	0 (0.0%)
Endoleak type III	0 (0.0%)	0 (0.0%)	0 (0.0%)

EVAR: endovascular abdominal aortic aneurysm repair.

**Table 3:** Technical details. Technical success and intra-operative information of the total cohort, as well as per type of repair.

Intra-Operative Details	Total Cohort (88 Patients)	Standard EVAR (77 Patients)	Parallel Graft EVAR (11 Patients)
Technical success	87 (98.8%)	77 (100.0%)	10 (90.9%)
Death on table	9 (10.2%)	9 (11.7%)	0 (0.0%)
Bifurcated EVAR	62 (70.5%)	51 (66.2%)	11 (100.0%)
AUI device	26 (29.5%)	26 (33.8%)	0 (0.0%)
Single parallel graft	-	-	1 (9.1%)
Double parallel grafts	-	-	7 (63.6%)
Triple parallel grafts	-	-	3 (27.3%)
Local and MAC anesthesia	56 (63.6%)	50 (64.9%)	6 (54.5%)
General anesthesia	32 (36.4%)	27 (35.1%)	5 (45.5%)
Transfusion	32 (36.4%)	28 (36.4%)	4 (36.4%)
Duration of operation (min)	182 ± 57	170 ± 63	248 ± 39

AUI: aorto-uni-iliac; EVAR: endovascular abdominal aortic aneurysm repair; MAC: monitored anesthesia care.

**Table 4:** Early outcomes. Thirty-day outcomes in patients managed with the Endurant endograft for a ruptured abdominal aortic aneurysm.

Early Outcomes	Total Cohort (88 Patients)	Standard EVAR (77 Patients)	Parallel Graft EVAR (11 Patients)
Mortality	42 (47.7%)	39 (50.6%)	3 (27.3%)
On-table mortality	9 (10.25)	9 (11.7%)	0 (0.0%)
Myocardial infarction	1 (1.1%)	1 (1.3%)	0 (0.0%)
Dialysis dependent AKI	3 (3.4%)	2 (2.6%)	1 (9.1%)
Respiratory insufficiency	2 (2.3%)	2 (2.6%)	0 (0.0%)
Multiorgan failure	27 (30.7%)	25 (32.5%)	2 (18.2%)
Reinterventions	3 (3.4%)	3 (3.9%)	0 (0.0%)
Endoleak type Ia	1 (1.1%)	1 (1.3%)	0 (0.0%)
Endoleak type Ib	0 (0.0%)	0 (0.0%)	0 (0.0%)
Endoleak type III	0 (0.0%)	0 (0.0%)	0 (0.0%)
Access related	2 (2.3%)	2 (2.6%)	0 (0.0%)

AKI: acute kidney injury; EVAR: endovascular abdominal aortic aneurysm repair.

## Data Availability

Data are available upon reasonable request from the corresponding author.

## Supplementary Materials

The following supporting information can be downloaded at https://www.mdpi.com/article/10.3390/jcm13175282/s1, Figure S1: Patient recruitment flow.

## References

[1] Laine M.T., Laukontaus S.J., Kantonen I., Venermo M. (2016). Population-based study of ruptured abdominal aortic aneurysm. Br. J. Surg..

[2] Menges A.L., DOria M., Zimmermann A., Dueppers P. (2023). Ruptured abdominal aorto-iliac aneurysms: Diagnosis, treatment, abdominal compartment syndrome, and role of simulation-based training. Semin. Vasc. Surg..

[3] D’Oria M., Gunnarsson K., Wanhainen A., Mani K. (2023). Long-term survival after repair of ruptured Abdominal Aortic Aneurysms is Improving Over Time—Nationwide Analysis During Twenty-four Years in Sweden (1994–2017). Ann. Surg..

[4] Fairman A.S., Wang G.J. (2020). Endovascular Treatment of Ruptured Abdominal Aortic Aneurysms. Semin. Interv. Radiol..

[5] Bruen K.J., Feezor R.J., Daniels M.J., Beck A.W., Lee W.A. (2011). Endovascular chimney technique versus open repair of juxtarenal and suprarenal aneurysms. J. Vasc. Surg..

[6] Le Houérou T., Fabre D., Alonso C.G., Brenot P., Bourkaib R., Angel C., Amsallem M., Haulon S. (2018). In Situ Antegrade Laser Fenestrations During Endovascular Aortic Repair. Eur. J. Vasc. Endovasc. Surg..

[7] Jernigan E.G., Tran N.N., Qato K., Giangola G., Carroccio A., Conway A.M. (2021). Outcomes of chimney/snorkel endovascular repair for symptomatic and ruptured abdominal aortic aneurysms. J. Vasc. Surg..

[8] Egorova N., Giacovelli J., Greco G., Gelijns A., Kent C.K., McKinsey J.F. (2008). National outcomes for the treatment of ruptured abdominal aortic aneurysm: Comparison of open versus endovascular repairs. J. Vasc. Surg..

[9] Mwipatayi B.P., Faraj J., Oshin O., Fitridge R., Wong J., Schermerhorn M.L., Becquemin J.-P., Boeckler D., Riambau V., Teijink J.A. (2021). Endurant stent graft demonstrates promising outcomes in challenging abdominal aortic aneurysm anatomy. J. Vasc. Surg..

[10] Georgiadis G.S., Schoretsanitis N., Argyriou C., Nikolopoulos E., Kapoulas K., Georgakarakos E.I., Ktenidis K., Lazarides M.K. (2023). Long-term outcomes of the Endurant endograft in patients undergoing endovascular abdominal aortic aneurysm repair. J. Vasc. Surg..

[11] Taneva G.T., Criado F.J., Torsello G., Veith F., Scali S.T., Kubilis P., Donas K.P., Dalman R.L., Tran K., Lee J. (2020). Results of chimney endovascular aneurysm repair as used in the PERICLES Registry to treat patients with suprarenal aortic pathologies. J. Vasc. Surg..

[12] Oliveira-Pinto J., Soares-Ferreira R., Oliveira N.F., Gonçalves F.M.B., Hoeks S., Van Rijn M.J., Raa S.T., Mansilha A., Verhagen H.J. (2020). Comparison of midterm results of endovascular aneurysm repair for ruptured and elective abdominal aortic aneurysms. J. Vasc. Surg..

[13] von Elm E., Altman D.G., Egger M., Pocock S.J., Gøtzsche P.C., Vanderbroucke J.P., Strobe Initiative (2014). The Strengthening the Reporting of Observational Studies in Epidemiology (STROBE) Statement: Guidelines for reporting observational studies. Int. J. Surg..

[14] Nana P., Spanos K., Kouvelos G., Arnaoutoglou E., Giannoukas A., Matsagkas M. (2023). Conical Aortic Neck as a Predictor of Outcome after Endovascular Aneurysm Exclusion: Midterm Results. Ann. Vasc. Surg..

[15] Wanhainen A., Van Herzeele I., Goncalves F.B., Montoya S.B., Berard X., Boyle J.R., D’oria M., Prendes C.F., Karkos C.D., Kazimierczak A. (2024). Editor’s Choice—European Society for Vascular Surgery (ESVS) 2024 Clinical Practice Guidelines on the Management of Abdominal Aorto-Iliac Artery Aneurysms. Eur. J. Vasc. Endovasc. Surg..

[16] Pitros C., Mansi P., Kakkos S. (2022). Endografts for the treatment of abdominal aortic aneurysms with a hostile neck anatomy: A systematic review. Front. Surg..

[17] Kouvelos G., Spanos K., Nana P., Mpatzalexis K., Rousas N., Mpareka M., Arnaoutoglou E., Giannoukas A., Matsagkas M. (2020). Endovascular treatment of ruptured pararenal abdominal aortic aneurysm using the Chimney technique. Hell. J. Vasc. Endovasc. Surg..

[18] Chaikof E.L., Blankensteijn J.D., Harris P.L., White G.H., Zarins C.K., Bernhard V.M., Matsumura J.S., May J., Veith F.J., Fillinger M.F. (2002). Reporting standards for endovascular aortic aneurysm repair. J. Vasc. Surg..

[19] Oderich G.S., Forbes T.L., Chaer R., Davies M.G., Lindsay T.F., Mastracci T., Singh M.J., Timaran C., Woo E.Y. (2021). Reporting standards for endovascular aortic repair of aneurysms involving the renal-mesenteric arteries. J. Vasc. Surg..

[20] Antoniou G.A., Antoniou S.A., Torella F. (2020). Editor’s Choice—Endovascular vs. Open Repair for Abdominal Aortic Aneurysm: Systematic Review and Me-ta-analysis of Updated Peri-operative and Long Term Data of Randomised Controlled Trials. Eur. J. Vasc. Endovasc. Surg..

[21] Hinchliffe R.J., Bruijstens L., MacSweeney S.T.R., Braithwaite B.D. (2006). A randomised trial of endovascular and open surgery for ruptured abdominal aortic aneurysm—Results of a pilot study and lessons learned for future studies. Eur. J. Vasc. Endovasc. Surg..

[22] van Beek S., Vahl A., Wisselink W., Reekers J., Legemate D., Balm R. (2015). Midterm re-interventions and survival after endovascular versus open repair for ruptured abdominal aortic aneurysm. Eur. J. Vasc. Endovasc. Surg..

[23] IMPROVE Trial Investigators (2017). Comparative clinical effectiveness and cost-effectiveness of an endovascular strategy versus open repair for ruptured abdominal aortic aneurysm: 3 year results of the IMPROVE randomised trial. BMJ.

[24] Desgranges P., Kobeiter H., Katsahian S., Bouffi M., Gouny P., Favre J.P., Alsac J.M., Sobocinski J., Julia P., Alimi Y. (2015). Editor’s choice—ECAR (Endovasculaire ou Chirurgie dans les Anevrysmes aorto-iliaques Rompus): A french randomized controlled trial of endovascular versus open surgical repair of ruptured aorto-iliac aneurysms. Eur. J. Vasc. Endovasc. Surg..

[25] Sweeting M.J., Ulug P., Balm R., Desgranges P., Powell J.T. (2015). Individual-patient meta-analysis of three randomized trials comparing endovascular versus open repair for ruptured abdominal aortic aneurysm. Br. J. Surg..

[26] Daviú-Molinari T., Choi J.C.-B., Roberts M.-C., Faridmoavear E., Sharath S.E., Kougias P. (2024). In-hospital Mortality Risk after Endovascular and Open Aortic Aneurysm Repairs for Ruptured Abdominal Aortic Aneurysms. J. Vasc. Surg..

[27] D’oria M., Scali S.T., Neal D., DeMartino R., Beck A.W., Mani K., Lepidi S., Huber T.S., Stone D.H. (2022). Center volume and failure to rescue after open or endovascular repair of ruptured abdominal aortic aneurysms. J. Vasc. Surg..

[28] Accarino G., De Vuono F., Accarino G., Fornino G., Puca A.E., Fimiani R., Parrella V., Savarese G., Furgiuele S., Vecchione C. (2024). Endurant Stent Graft for Treatment of Abdominal Aortic Aneurysm Inside and Outside of the Instructions for Use for the Proximal Neck: A 14-Year, Single-Center Experience. J. Clin. Med..

[29] Le Houérou T., Álvarez-Marcos F., Gaudin A., Bosse C., Costanzo A., Vallée A., Haulon S., Fabre D. (2023). Midterm Outcomes of Antegrade In Situ Laser Fenestration of Polyester Endografts for Urgent Treatment of Aortic Pathologies Involving the Visceral and Renal Arteries. Eur. J. Vasc. Endovasc. Surg..

[30] Tsilimparis N., Gouveia e Melo R., Tenorio E.R., Scali S., Mendes B., Han S., Schermerhorn M., Adam D.J., BMalas M., Farber M. (2024). Multicenter Study on Physician-Modified Endografts for Thoracoabdominal and Complex Abdominal Aortic Aneurysm Repair. Circulation.

[31] Nana P., Spanos K., Jakimowicz T., Torrealba J.I., Jama K., Panuccio G., Rohlffs F., Kölbel T. (2023). Urgent and emergent repair of complex aortic aneurysms using an off-the-shelf branched device. Front. Cardiovasc. Med..

[32] Fazzini S., Turriziani V., Tonidandel L., Vona S., Marchetti A.A., Ippoliti A., Austermann M., Torsello G. (2024). Protagoras 3.0: Feasibility of current fenestrated endografts and chimney technique for complex abdominal aortic aneurysms. J. Cardiovasc. Surg..

[33] Keschenau P.R., Beropoulis E., Gombert A., Jacobs M.J., Torsello G., Austermann M., Kotelis D., Donas K.P. (2021). The role of surgical and total endovascular techniques in the treatment of ruptured juxtarenal aortic aneurysms. Vasa.

[34] Usai M.V., Beropoulis E., Fazzini S., Avranas K., Khatatba Y., Pitoulias A., Taneva G.T., Austermann M.J., Donas K.P. (2024). Early multicentric outcomes of the on-label and CE-marked combination of the Endurant with the Radiant chimney graft for the chimney endovascular aortic repair (EnChEVAR): The LaMuR Registry. Cardiovasc Surg..

[35] Nana P., Spanos K., Apostolidis G., Haulon S., Kölbel T. (2024). Systematic review and meta-analysis of fenestrated or branched devices after previous open surgical aortic aneurysm repair. J. Vasc. Surg..

[36] Nana P., Kölbel T., Behrendt C.-A., Kouvelos G., Giannoukas A., Haulon S., Spanos K. (2023). Systematic review of reintervention with fenestrated or branched devices after failed previous endovascular aortic aneurysm repair. J. Vasc. Surg..

[37] Ozdemir-van Brunschot D.M., Torsello G.B., Bernandini G., Litterscheid S., Torsello G.F., Beropoulis E. (2023). Long-term Results of Angulated Versus Hyperangulated Neck in Endovascular Aneurysm Repair With Endurant Endoprosthesis. J. Endovasc. Ther..

[38] Taneva G.T., Donas K.P., Torsello G.B., Seifarth H., Marques de Azevedo F., Austermann M., Torsello G.F. (2019). In Vitro Evaluation of Balloon-Expandable Chimney Grafts in the Renal Arteries Combined With the Endurant Endograft. J. Endovasc. Ther..

[39] Jabr A.B., Lindblad B., Dias N., Resch T., Malina M. (2015). Efficacy and durability of the chimney graft technique in urgent and complex thoracic endovascular aortic repair. J. Vasc. Surg..

[40] Lindbald B., Bin Jabr A., Holst JMalina M. (2015). Chimney Grafts in Aortic Stent Grafting: Hazardous or Useful Technique? Systematic Review of Current Data. Eur. J. Vasc. Endovasc. Surg..

[41] Song Q., Guo Y., Huo Z., Wang M., Sun X., Zhou Z., Bi C., Dong D., Gao P., Wu X. (2024). Analysis of high-risk factors and mortality prediction of ruptured abdominal aortic aneurysm. Ann. Vasc. Surg..

[42] Gallitto E., Faggioli G., Austermann M., Kölbel T., Tsilimparis N., Dias N., Melissano G., Simonte G., Katsargyris A., Oikonomou K. (2024). Urgent endovascular repair of juxta/para-renal aneurysm by off-the-shelf multibranched endograft. J. Vasc. Surg..

[43] Spanos K., Volakakis G., Kouvelos G., Haidoulis A., Dakis K., Karathanos C., Stamatiou G., Arnaoutoglou E., Matsagkas M., Giannoukas A. (2024). Transition from Open Repair to Endovascular Aneurysm Repair for Rupture Aortic Aneurysms throughout a 16-Year Period of Time in a Single Tertiary Center. Ann. Vasc. Surg..

[44] Schutt J., Bohr N.L., Cao K., Pocivavsek L., Milner R. (2024). Social Determinants of Health Factors and Loss-To-Follow-Up in the Field of Vascular Surgery. Ann. Vasc. Surg..

[45] Powell J.T., Sweeting M.J., Ulug P., Thompson M.M., Hinchliffe R.J., IMPROVE Trial Investigators (2018). Editor’s Choice—Re-interventions After Repair of Ruptured Abdominal Aortic Aneurysm: A Report From the IMPROVE Randomised Trial. Eur. J. Vasc. Endovasc. Surg..

[46] Li S.R., Phillips A.R., Reitz K.M., Mikati N., Brown J.B., Tzeng E., Makaroun M.S., Guyette F.X., Liang N.L. (2024). Hypertension during transfer is associated with poor outcomes in unstable patients with ruptured abdominal aortic aneurysm. J. Vasc. Surg..

