# Supplementary material for: Endovascular Repair of Ruptured Abdominal Aortic Aneurysms Using the Endurant™ Endograft
Source: J Clin Med. 2024 Sep 6;13(17):5282. doi: 10.3390/jcm13175282 (PMC11396500; doi:10.3390/jcm13175282)
Supplement: Supplementary file 1 [file jcm-13-05282-s001.zip › jcm-3174184-supplementary.pdf]

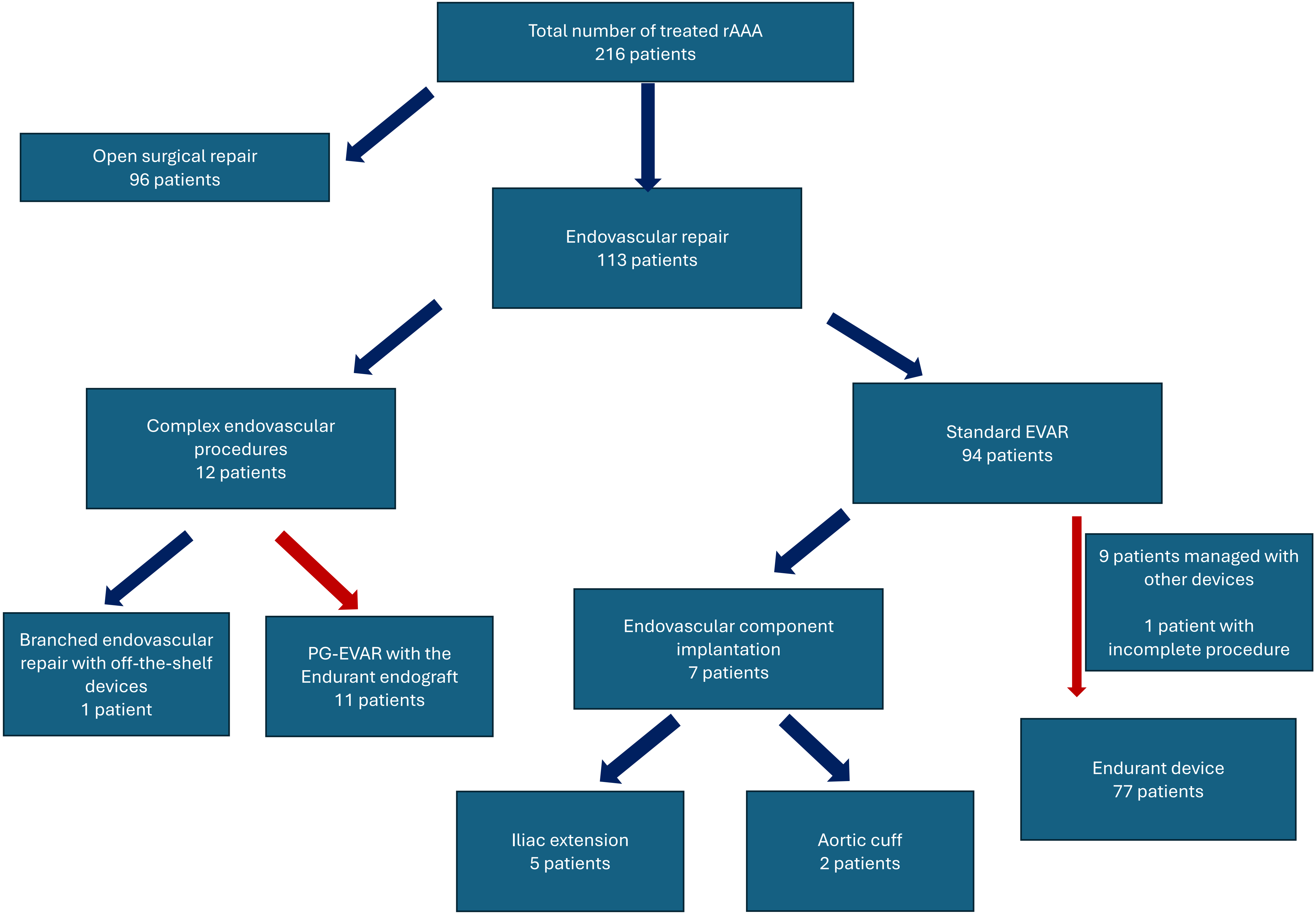
Total number of treated rAAA
216 patients
Open surgical repair
96 patients
Endovascular repair
113 patients
Complex endovascular procedures
12 patients
Standard EVAR
94 patients
Branched endovascular repair with off-the-shelf devices
1 patient
PG-EVAR with the Endurant endograft
11 patients
Endovascular component implantation
7 patients
9 patients managed with other devices
1 patient with incomplete procedure
Endurant device
77 patients
Iliac extension
5 patients
Aortic cuff
2 patients
